# Recent insights into the implications of UGDH mutations for human developmental disease

**DOI:** 10.1042/BST20253083

**Published:** 2025-08-28

**Authors:** Hali Harwood, Brenna M. Zimmer, Asher R. Utz, Joseph J. Barycki, Melanie A. Simpson

**Affiliations:** Department of Molecular and Structural Biochemistry, North Carolina State University, Raleigh, NC, 27695, U.S.A.

**Keywords:** cardiac valve defects, congenital disorders of glycosylation, developmental and epileptic encephalopathy, dystroglycanopathy, epilepsy, UDP-glucose dehydrogenase

## Abstract

Congenital disorders of glycosylation are a significant underlying cause of developmental and epileptic encephalopathy (DEE). A subset of these DEE cases results from biallelic variants in the unique, essential gene encoding UDP-glucose dehydrogenase (UGDH). The UGDH enzyme catalyzes two successive NAD^+-^ dependent oxidation reactions to convert the C6 hydroxyl of UDP-glucose to a carboxylate, generating the UDP-glucuronate product. This product is required for three critical reactions that generate: (1) hyaluronan, (2) secreted and cell surface proteoglycans, and (3) glucuronide conjugates for cellular detoxification. UGDH polymorphisms are not frequently observed as they are largely deleterious. However, a number of UGDH variants have been reported and characterized as causative agents of congenital defects in cardiac valve and brain development, and most recently of dystroglycanopathy. The effects of these mutations, clinically and at the molecular level, are summarized and discussed in this review.

## Introduction

Developmental and epileptic encephalopathy (DEE) is defined by the onset of severe epileptic seizures in infancy or childhood [1]. The specific timing of onset and the severity of symptoms may vary. There are at least 20 known genes in which allele variants have been shown to cause DEE, which is a subset of the larger category of congenital disorders of glycosylation [2]. These are typically autosomal recessive in the pattern of inheritance. Mutational analysis of one of the causative genes, UDP-glucose dehydrogenase (UGDH), was first reported in 2020 [3]. In this report, 23 clinically identified variants were listed in association with a spectrum of conditions ranging from developmental delay and congenital microcephaly (CM) unaccompanied by epileptic seizures to early infantile or neonatal epileptic encephalopathy [3]. This spectrum of disorders is now collectively referred to as UGDH-related disorders and/or Jamuar Syndrome, in recognition of the causative gene and the first clinician to coalesce research around the disorder, respectively. In contrast with other causative genes, UGDH is not yet routinely sequenced as an underlying cause of epilepsy or developmental delay, so the frequency of its variants is likely underestimated at approximately one in two million.

UGDH-related disorders are heterogeneous in their manifestation but, in general, are devastating diseases that affect lifespan and quality of life, often requiring constant care. As congenital disorders of glycosylation, UGDH-related disorders are autosomal recessive, with each parent bearing an affected allele that may encode the same or different mutations. The disorder known as Jamuar Syndrome occurs when both alleles encode a variant of UGDH that is defective. This is also true of the recently identified variants associated with dystroglycanopathies that have central nervous system (CNS) involvement [4]. However, two UGDH variants were previously reported to give rise to congenital cardiac valve defects when present in individuals heterozygous for the wildtype (WT) and UGDH variant allele [5]. The characterization of a defective variant is a critical component of predicting disease severity. Multiple factors including intrinsic stability and function of the variant, other genetic background variables, idiopathic factors, and individual environmental exposures can affect the manifestation of disease in patients with UGDH variants.

### Role of UGDH in normal and pathological conditions

**Figure 1:**
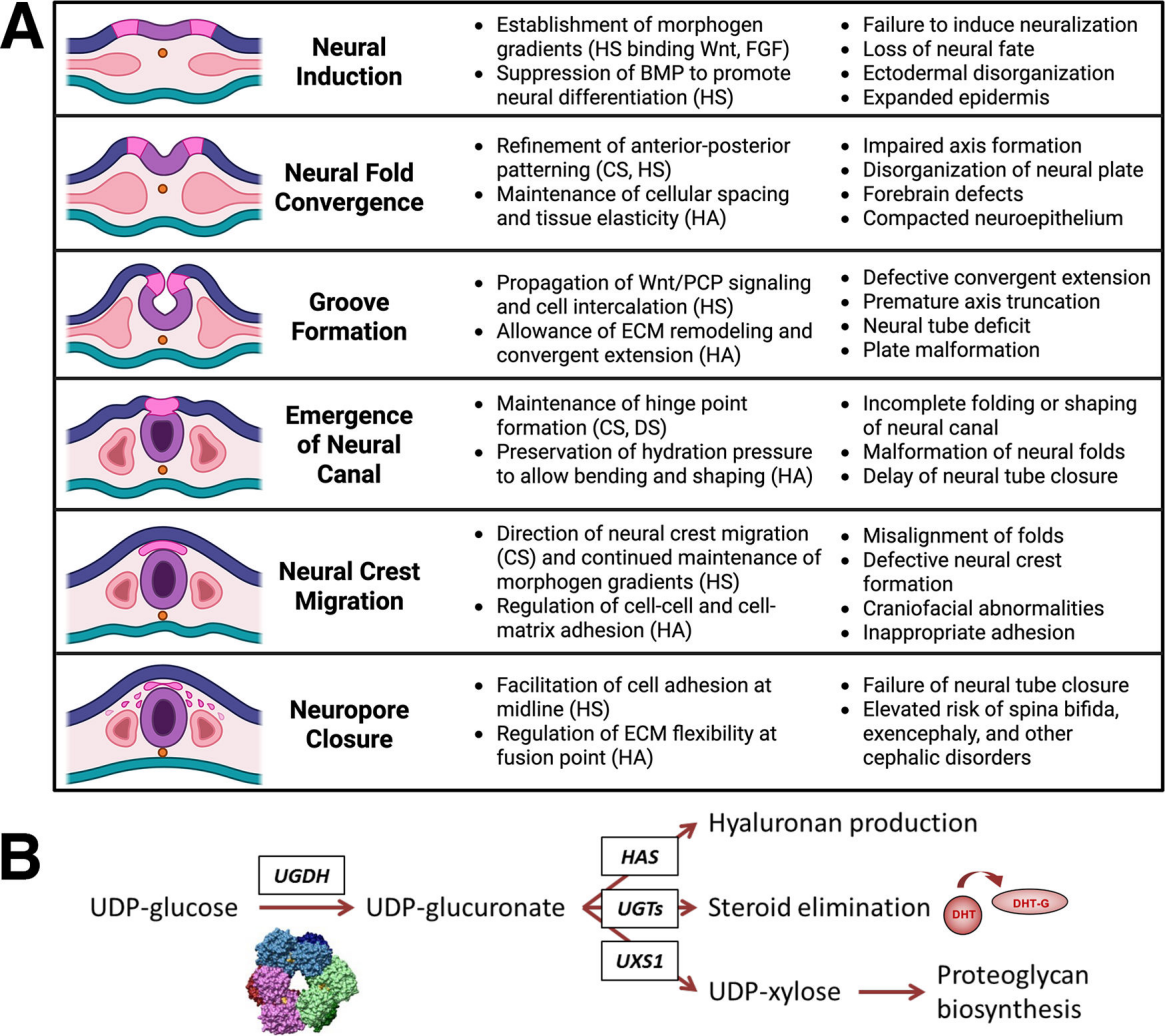
Importance of UGDH for human development. (**A**) Schematic (left column) and summary of stages in early development that are glycosaminoglycan dependent (center column) and consequences of UGDH deficiency (right column). Created in https://BioRender.com. (**B**) Substrate and product of UGDH catalysis and schematic of downstream pathways. BMP, bone morphogenetic protein; CS, chondroitin sulfate; DS, dermatan sulfate; HA, hyaluronan; HS, heparan sulfate; DHT, dihydrotestosterone; DHT-G, dihydrotestosterone-glucuronide; FGF, fibroblast growth factor; HAS, hyaluronan synthase; PCP, planar cell polarity; UGT, UDP-glucuronosyltransferase; UXS, UDP-xylose synthase; Wnt, Wingless/Integrated.

UGDH is a unique essential gene that is conserved across species such as humans, mice, zebrafish, and frogs [6–9]. In model organisms, its deletion is embryonic lethal, typically due to a halt in cardiovascular development at early embryonic stages. UGDH catalyzes the NAD^+^-dependent formation of UDP-glucuronate from UDP-glucose [10–12]. UDP-glucuronate supports developmentally timed events that require rapid production of hyaluronan (HA), an extracellular glycosaminoglycan (GAG) critical for tissue morphogenesis, as well as the glycan initiation and extension necessary for all proteoglycans, which are critical throughout development. Figure 1A provides a schematic overview of early stages in CNS development that rely on the product of UGDH for correct expression and abundance of morphogens such as bone morphogenetic protein, and GAGs including HA, heparan sulfate, chondroitin sulfate, and dermatan sulfate. Essential processes and the consequences of GAG insufficiency are compiled and summarized in the figure from several excellent reviews [13–23]. Importantly, UGDH also provides the UDP-glucuronate precursor for glucuronidation, an essential phase II detoxification process active in embryogenesis and throughout life [24], which controls the effects of chemical exposure, as well as endogenous lipophiles and hormones (Figure 1B).

### Structural insights and functional implications of UGDH in development and disease

**Figure 2:**
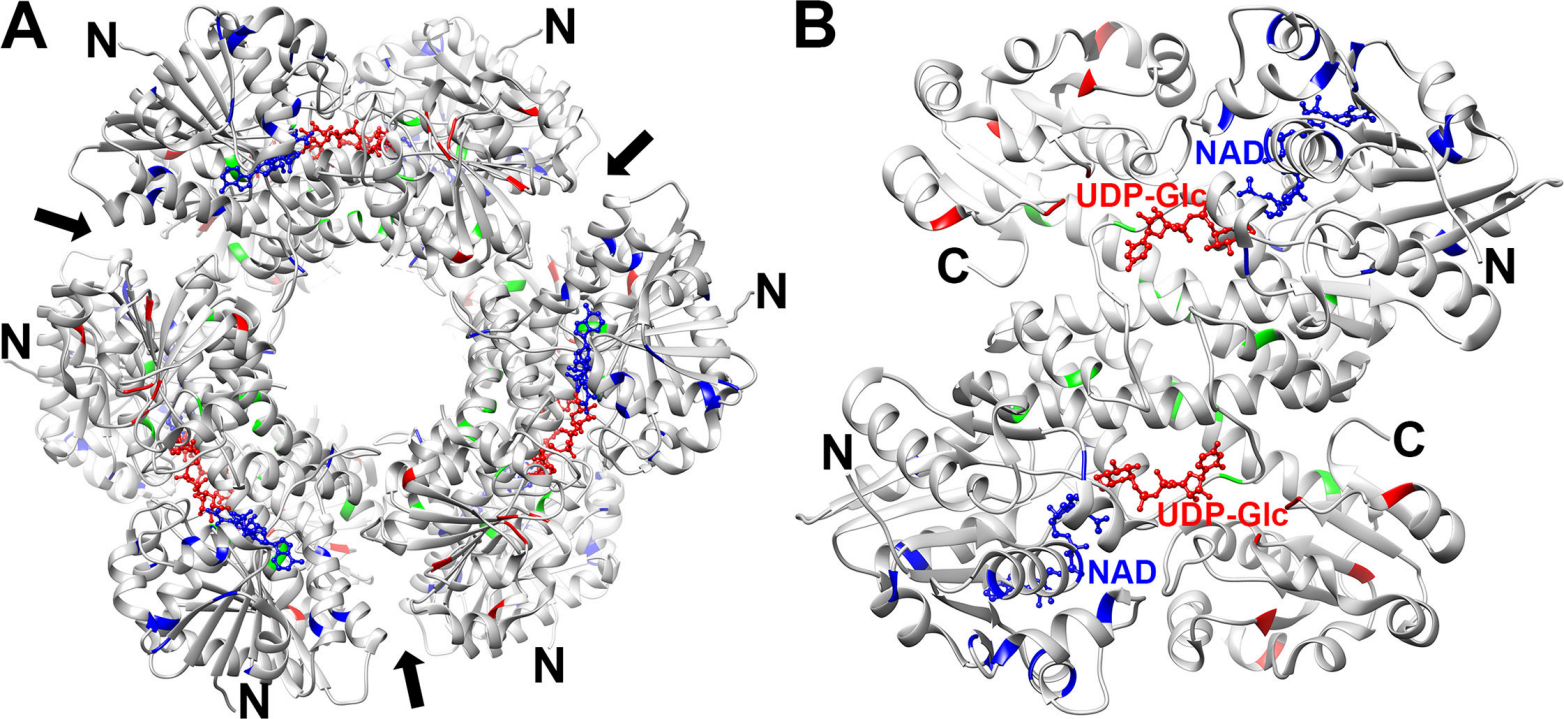
Ribbon diagram illustrating the position of UGDH variants known to cause UGDH-related disorders. (**A**) Overall ribbon of UGDH hexamer in grayscale with NAD variant positions indicated in blue (residues 5–212), UDP-glucose variants in red (residues 329–466), and central domain variants in green (213–323). NAD cofactor and UDP-glucose substrate are depicted in blue and red, respectively, and shown in ball and stick. The dimer–dimer interfaces are indicated with black arrows, and N termini for each of the six UGDH subunits that comprise the hexamer are designated with N; (**B**) the ribbon representation of a single dimer is rotated 90° horizontally to emphasize variants at the central domain of the dimeric unit.

Structural information on UGDH has been used extensively to guide predictions on intrinsic outcomes to the physical and functional properties of the enzyme. The crystal structures of UGDH in the absence of ligands and in the presence of substrate, product, cofactor, or as a holo complex containing both substrate/NADH and product/NAD^+^ have been solved to high resolution [25–30], so there is considerable insight available for modeling probable effects of the UGDH clinical variants. UGDH is described as a trimer of dimeric units, in which the monomers that comprise the dimeric unit are generally found to be inseparable in the purified enzyme without irreversible deleterious consequences [31,32]. Not surprisingly, variants do not typically occur in elements of structure important to the formation of the dimeric units, such as the regions of the core with lowest intrinsic mobility. Structural elements are designated according to those involved in shaping domains that support UDP-sugar substrate binding, NAD cofactor binding, dimer formation (central domain), dimer–dimer association, and the C-terminal intrinsically disordered tail (reviewed in [33]). The latter comprises residues 466–494, which are not visible in the electron density at a resolution that supports assignment of crystal co-ordinates for the atoms in that region. Most of the clinical variants associated with UGDH-related disorders are found in the elements of secondary structure that shape the so-called substrate or cofactor-binding domains, implying that truly disruptive mutations are not observed because they are deleterious (Figure 2). It is noteworthy that the variant residues depicted in the figure do not typically occur in proximity to the bound NAD cofactor or UDP-glucose substrate, nor in the dimer–dimer interface, but are generally peripheral and predicted to affect dynamic motion in the domains rather than eliminating direct binding contacts or catalytic residues.

- The first report in 2020 [3] provided a mechanistic basis for the occurrence of developmental disorders in patients with recessive UGDH variants. Seventeen patients with a total of 23 unique variants were examined in this work (Table 1). A subset of the variants was characterized using fibroblasts derived from patient biopsies, expression in a zebrafish model, and culture of cerebral organoids generated from patient fibroblasts induced for pluripotency and then allowed to differentiate. These studies established causality of the UGDH variants in disease. Consistently, the UGDH variants that appeared in affected patients had lower activity than the WT or heterozygous WT parents or siblings. All patient-derived cells had equivalent mRNA levels as expected, and most also had significant reductions in UGDH protein expression. When recreated in purified human UGDH protein, several of the point mutants had dramatically reduced activity and intrinsic stability, but in some cases, the effects were not as obvious and the explanations were more nuanced.

**Table 1:** Summary of UGDH variants with functional characterization

Allele variant		Clinical class	Functional characterization		
1	2		Cell culture or organism model	Purified UGDH protein	Ref
**NAD (cofactor)-binding domain (residues 5–212**)					
A44V	A44V	DEE	Fibroblast lysate activity negligible, UGDH expression reduced	rUGDH less stable to proteolysis and ≈3˚C lower Tm, stability partially recovered in holoenzyme, dimeric, reduced activity *in vitro* by ≈75%	[3], [34]
A44V	R65*	DEE	R65 premature truncation assumed not to yield protein, only A44V makes full-length UGDH	As above	[3]
A82T	A82T	DEE	Fibroblast lysate activity negligible; UGDH expression and HA production reduced. Cerebral organoid model: 50% reduced size relative to WT or heterozygous, impaired neuronal differentiation, reduced detection of PAX6/TBR2 early/intermediate neuronal markers	rUGDH highly unstable to heat and proteolysis, reduced activity *in vitro* by 80%	[3]
Y14C	S72P	DEE	Fibroblast lysate activity negligible, UGDH expression reduced, 50% reduced cerebral organoid size	No purified protein data	[3]
A24V	R135Q	CM	Variants not modeled, but UGDH knockout in zebrafish produced changes in head size and CNS that recapitulate human CM.	rUGDH: both variants had reduced activity; A24V had reduced thermal stability	[35]
L57I	T244K	CM	Not modeled	Both mutants had reduced activity and thermal stability	[35]
R141C	WT	CVD	Could not rescue atrioventricular septum and cardiovascular formation in UGDH null zebrafish	Activity 60% reduced, dimeric, lower stability, reduced T_1/2_ conferred to WT UGDH	[5]
**UDP-sugar binding domain (residues 329–466**)					
Y367C	R65*	DEE	Fibroblast lysate activity negligible, UGDH expression reduced; in cerebral organoid model: 50% reduced size relative to WT or heterozygous, no change in neuron function but impaired differentiation, reduced detection of PAX6/TBR2 early/intermediate neuronal markers	No purified protein data	[3]
R393W	A410S	DEE	Fibroblast lysate activity negligible, UGDH expression unaffected	No purified protein data	[3]
E416D	WT	CVD	In zebrafish, no atrioventricular septum formed in UGDH morpholino knockdown, UGDH E416D transcripts could not rescue	rUGDH: activity similar but reduced T_1/2_ that was conferred to WT UGDH	[5]

CM, congenital microcephaly. CVDs, cardiac valve defects. DEE, developmental and epileptic encephalopathy.

### Variants characterized for functional impacts

A number of UGDH variants have been characterized in addition to those in the 2020 inaugural report, and a summary of all outcomes is presented here to integrate the available findings at the level of clinical, *in silico*, structural, and functional analysis. Once clinically identified by genome or exome sequencing and classical genetic mapping, approaches to confirm and validate a causative function of the UGDH variant include computational model prediction, purified protein analysis, and the recapitulation of the variant in cells and model organisms. Below we discuss first the UGDH variants for which functional data are present (listed in Table 1) and then provide detailed structural insights for the large number of additional variants (Table 2). For a comprehensive discussion of clinical manifestations in each patient, please see the literature summaries in [4,5,34,37,38].

**Table 2:** Summary of UGDH variant in silico and structural predictions

Allele variant		Clinical class	Structural insights^1^	Ref
1	2			
**NAD (cofactor)-binding domain (residues 5–212)**				
A24T	P175A	DEE	Ala24 is located in the folding core of the NAD-binding domain and H bonds to side chain amide of N68, which is in a constrained turn between a helix and beta strand. Thr substitution will disrupt folding. Pro175 is in a turn between a helix and a strand in the NAD-binding domain and also co-ordinates to Lys207 in an adjacent loop. Substitution to Ala probably disrupts placement of the strand following the helix.	[3]
I42T	Y356^*^	DEE	Ile42 is in a helix with Arg41, which co-ordinates the bridging phosphates and ribose hydroxyl of NAD; substitution to Thr could introduce a polar functional group into a hydrophobic environment or could affect stability/placement of the helix for NAD binding. Tyr356* is a premature truncation and probably does not fold.	[3]
A44V	N68Y	DEE	Val44 sterically interferes with P51, disrupts secondary structure elements adjacent to the bound NAD cofactor, including Arg41, which co-ordinates the bridging phosphates and ribose hydroxyl of NAD. Asn68 is located in a turn between helix and strand and positions the beginning of an adjacent beta strand, H bonds carbonyl oxygen of Ala24 in the folding core. Substitution to Tyr sterically disrupts a tightly packed region of the NAD domain.	[36]
R102Q	c.265–6 C > G	DG	Arg 102 is in a relatively surface-exposed loop in contact with solvent: substitution with Gln may be tolerated, but because the loop stabilizes both the NAD domain and the dimer–dimer interaction domain, activity is probably compromised.	[4]
R102W	R65^*^	Global psychomotor delay, no seizures	Arg 102 is in a loop as described above: substitution with Trp would place a hydrophobic residue in solvent-exposed position, which may be unfavorable to stability of folding. In addition, the disruption of the NAD domain and hexamer formation is likely contributing to reduced activity.	[37]
R135W	R65^*^	DEE	Arg 135 H bonds backbone carbonyl of T244 in adjacent subunit of homodimer, Trp would likely not be able to substitute for that critical H bond so the dimeric unit would not be stable, likely a folding mutant.	[37]
I116T	R443H	DEE	Ile 116 is in the tightly packed hydrophobic core of the domain, including V85, V126, I10, I79, L154, and F144. This core directly abuts the adenine moiety of bound NAD. Hydrophilic Thr is not likely to be tolerated, and the folded structure would be disrupted. Arg 443 is in the UDP-sugar binding domain, guanidino co-ordinates carboxylates of D247 and D446, and H bonds backbone carbonyl of I462, proximal to T244. Critical for domain stability, monomer–monomer stability, and likely for overall folding.	[3]
**Central domain (residues 213–323**)				
A44V	E217D	DEE	A44 as above. Glu217 co-ordinates the backbone amides of Arg135 and Arg137. The shortened chain length of Asp relative to Glu disrupts positioning of Arg135 at the start of the adjacent helix, affecting folding.	[3]
I255T	G271R	DEE	Ile 255 is in a hydrophobic domain critical for dimer formation, near F226 and A222 on the adjacent subunit, disruption will compromise via packing defects. Gly 271 is in a tightly packed loop of the protein core. Ser 269 H bonds backbone amide, Ser 349 H bonds backbone carbonyl of 271. Due to tight packing and two proximal Args (R442 and R311), Arg would be both sterically and electrostatically incompatible.	[3]
V303I	V303I	DEE	Nucleotide substitution affects RNA splice site. Val to Ile as a substitution at 303 does not appear to be intrinsically disruptive. Probably leads to premature termination by affecting message splicing.	[3]
V303I	R443H	DEE	Val303 as above. His443, as above, likely produces a folding mutant.	[3]
M306V	E155^*^	DEE	Met 306 is in the monomer–monomer interacting domain, critical for forming the dimeric unit. Disruption will produce poorly folded protein through packing defects. Glu155* is prematurely truncated.	[3]
R317Q	R317Q	DEE	Arg 317 in helix that mediates dimer–dimer interaction, facing internally. Guanidino salt bridges E460 carboxylate to stabilize helix interaction with adjacent beta strand. Substitution to Gln could allow H bonding to the E460 carboxylate, which may be tolerated structurally but produce suboptimal dimer–dimer association, thereby potentially reducing activity.	[3,38,39]
**UDP-sugar binding domain (residues 329–466**)				
V303I	R443H	DEE	V303 no protein. Arg 443 is in the UDP-sugar binding domain, guanidino co-ordinates carboxylates of D247 and D446, H bonds backbone carbonyl of I462, proximal to T244. Critical for domain stability, monomer–monomer stability, and likely for overall folding.	[3]
R442W	R443H	DEE	Arg 442 guanidino co-ordinates the backbone carbonyl of C414 in adjacent loops of the UDP-sugar domain. The guanidino also directly co-ordinates the 2′ hydroxyl of the UDP ribose moiety of the substrate. Substrate binding via the ribose moiety is partially contributed by the backbone carbonyl of F338. Therefore, substitution with Trp likely affects substrate binding either directly or indirectly.	[3]
H449R	E155^*^	DEE	His 449 co-ordinates a bound water, which positions three different elements of structure through carboxylates of D440, D446, and hydroxyl of T461. Substitution will disrupt local structure of the UDP-sugar binding domain and place a positive charge adjacent to other positively charged Args. Because D440 positions R442, which directly binds the UDP-sugar, substitution of Arg likely disrupts substrate binding.	[3]

1 Discussed in text

CM, congenital microcephaly. DEE, developmental and epileptic encephalopathy. DG, dystroglycanopathy.

#### A44V

Several patients with clinically designated DEE were found to bear the UGDH A44V variant, which is located in the domain that spans amino acid residues 5–212 and constitutes the NAD cofactor-binding domain. Fibroblasts derived from a homozygous patient biopsy were characterized and found to have significantly reduced UGDH protein expression and negligible UGDH activity in whole-cell lysates relative to fibroblasts derived from siblings and parents that were homozygous or heterozygous for WT UGDH, respectively [3]. When recreated in human UGDH purified protein, the resulting UGDH A44V enzyme was dimeric and could only partially be stabilized to the fully active hexameric conformation. Intrinsic stability of the enzyme was reduced, and it was only about 25% as active as the WT UGDH. Loss of cofactor binding is the probable outcome of the A44V variant. Inspection of the structure shows that a Val44 substitution sterically interferes with Pro51, which is predicted to disrupt secondary structure elements adjacent to the bound NAD cofactor, including Arg41, which co-ordinates the bridging phosphates and ribose hydroxyl of NAD.

#### A82T

Ala82 is adjacent to the core beta sheet that forms the NAD-binding site. The substitution of another residue at this location is not predicted to be sterically compatible with protein folding. Fibroblasts were cultured from a biopsied DEE patient found homozygous for the UGDH A82T allele [3]. Activity in the fibroblast lysates was negligible, UGDH protein expression was very low, and the fibroblasts produced significantly less HA than heterozygous or homozygous UGDH WT fibroblasts. These fibroblasts were used to generate cerebral organoids, which could be used to model and reveal CNS developmental defects. This was done in lieu of using an animal model because the zebrafish model of I331N, shown to lack UGDH expression [9], did not have CNS defects and was deemed unsuitable for DEE studies. Using the organoid model, it was possible to reconstitute neuronal differentiation and show that the A82T variants gave rise to 50% smaller organoids that were deficient in proliferating cells expressing PAX6 and TBR2, markers of early and intermediate neuronal differentiation. Despite the lower presence of specific neuronal subsets, neuron function was unaltered, suggesting the loss of UGDH activity in early CNS development causes deficiencies in neuronal differentiation without affecting function.

#### Y14C/S72P

Fibroblasts derived from this heterozygous patient, bearing two different UGDH variant alleles, had significantly reduced UGDH activity and protein expression and produced similar deficiencies in cerebral organoid formation to the UGDH A82T homozygote [3]. In the UGDH structure, Tyr14 co-ordinates a network of bound waters that position Tyr53 and Gly343 through their backbone amides. Substitution of Tyr14 with Cys creates a cavity that could weaken the NAD-binding site by failing to stabilize positioning residues Glu165 and Arg346 (which directly co-ordinate NAD). Ser72 forms hydrogen bonds with the amide of Asn74 in a geometrically confined turn linking a beta strand to a helix. Ser72 substitution with Pro is likely destabilizing to the UGDH overall structure and disrupts folding.

#### A24V/R135Q and L57I/T244K

Two patients were recently identified with CM, in addition to DEE [35]. Both were heterozygous for new variants of UGDH. The patients were not biopsied, so the variants were recreated *in vitro* to test functional outcomes. All four purified UGDH variants had reduced enzymatic activity, and three of the four also had reduced intrinsic thermal stability, consistent with the disease-causing potential of the variant. Ala24 is located in the folding core of the NAD-binding domain, where it forms a hydrogen bond with the side chain amide of Asn68. Asn68 is in a turn of helix-turn-sheet where any substitution could disrupt folding. The side chain guanidino moiety of Arg135 forms a hydrogen bond with the backbone carbonyl of Thr244, an adjacent helix in the central domain of the neighboring dimer needed for stability among subunits. Glu217 in the same subunit also co-ordinates to the backbone of Arg135. If not positioned correctly by the substitution of Gln at 135, the NAD domain stability will be significantly affected. Leu57 is in a packed hydrophobic environment that may not sterically be able to accommodate any substitution, so Ile, although still hydrophobic, would be predicted to destabilize the overall structure, accounting for reduced enzymatic activity of ≈50%. Thr 244 is in the central domain, in a helix that packs alongside a helix of the adjacent subunit to form the dimeric core that does not separate without denaturation. The Thr244 side chain is wedged into the interface between subunits of the dimer, which is highly hydrophobic. The Thr hydroxyl is tolerated because it forms hydrogen bonds with several backbone elements to position helices. However, the Thr244 carbonyl is co-ordinated by Arg135 in the adjacent subunit, so the substitution with Lys is both sterically and electrostatically unfavorable in that region. Thus, all four of these UGDH mutants, although still potentially able to produce protein, are likely to have issues of misfolding and poor catalytic activity.

#### R141C and E416D

In a zebrafish screen for genes involved in heart development, UGDH was identified and validated as essential via the production of proteoglycan receptors involved in the sensing of growth factors and extracellular matrix (ECM)-based signals for morphogenesis [5]. Subsequently, variants of UGDH were sought in human patients with defects in heart development, and the role of heterozygous UGDH mutants was validated in congenital cardiac valve defects. Neither R141C nor E416D mRNA was able to rescue morpholino-suppressed UGDH knockdown in zebrafish, which failed to form the atrioventricular septum during embryonic cardiac morphogenesis and was ultimately lethal. *In vitro*, the respective variants recreated in purified UGDH were less catalytically active and less thermally stable, and this was found to affect the stability of WT UGDH as well, providing mechanistic insights into the effect of the mutants on human development.

#### Y367C/R65*

Fibroblasts derived from DEE patient biopsies bearing UGDH Y367C as a heterozygous combination with the prematurely truncated nonsense mutation of UGDH at position 65 had negligible UGDH expression and activity [3]. Similarly, the UGDH genotype in these cells was not able to support neuronal differentiation in the cerebral organoid model, yielding deficiencies in early and intermediate neuronal markers. Y367 is located at the end of a beta strand with its hydroxyl oriented toward the surface of the protein. Substitution of Cys at this site significantly reduces the steric and hydrophobic contacts with three adjacent secondary structure elements via proximal residues Pro401, Leu334, and Val420, likely affecting the folded stability of the UDP-sugar domain. Thus, this variant is probably creating a packing defect.

#### R393W/A410S

DEE patient-derived fibroblasts with this heterozygous UGDH allele combination were deficient in activity but expressed UGDH at levels comparable to the WT, which was unique among the variants characterized to date [3]. A structural inspection revealed that Arg393 is proximal to the dimer–dimer interface but is also solvent-exposed. Therefore, Trp substitution could be sterically tolerated if there were sufficient local contacts to shield its hydrophobicity. However, although substitution with Trp is likely tolerated, placing a Trp at this location is still unfavorable and probably alters the local tertiary structure to prevent dimer–dimer interaction and reduce catalytic activity. Ala410 is located in a hydrophobic pocket containing Phe437, Phe439, Leu322, Val412, and Ile331. Thus, the substituted Ser hydroxyl would likely not be tolerated well and would be expected to contribute to folding defects and/or local instability. This particular genotype is an example of two variants that could be relatively functional if not expressed together.

### Structural insights regarding UGDH variants not functionally characterized

UGDH variants that have not been functionally recreated in model organisms or recombinant purified UGDH protein have been analyzed *in silico,* and a summary of the predicted outcome of each variant based on the role of the original residue is given in Table 2. Here, we provide a general overview of the nature of these variants by domain (NAD, central, and UDP-sugar). In the NAD-binding domain, A24T, P175A, I42T, A44V, N68Y, R102W, and R135W all give rise to interactions that would destabilize and compromise the folded structure of the domain. This has been functionally demonstrated for A44V and would need to be individually determined for each of the other variants to be certain but strongly suggests that these variants are viable because they are substitutions generally compatible with synthesis and marginal activity of the resultant UGDH protein. However, because of the critical demand for abundant output of UDP-glucuronate at specific time points in development, suboptimal UGDH activity is not adequate to support key morphogenic events.

A specific example of the impact of partial UGDH activity on development is the variation at position 102, normally Arg. Variants were separately reported in a patient with multiple symptoms of developmental delay, but no seizures [37], and in two siblings with dystroglycanopathy, a subtype of muscular dystrophy, one of whom experienced seizures and the other who did not [4]. In the latter patients, the muscle structural protein α-dystroglycan was hypoglycosylated, likely due to deficiency in the UDP-glucuronate precursor for synthesis of its glycan chain and implying only partial UGDH activity. Both patient genotypes, R102W and R102Q, were heterozygous, with the other allele predicted to encode no protein. An inspection of the structural environment surrounding R102 reveals that both Trp and Gln variants would likely be tolerated and compatible with UGDH expression. However, because R102 positions a loop that communicates directly to the base of the NAD-binding domain and to the dimer–dimer interface, defects in both cofactor binding and hexamer integrity probably lead to lower activity.

Even more pronounced is the impact of variation in the central domain of the UGDH protein required for the integrity of the dimeric units. Since the dimeric unit is the minimum functional unit across species (exemplified by this form being the functional form in bacteria), it is not surprising that mutations to this domain fatally disrupt UGDH stability and therefore eliminate activity. As summarized in Table 2, all the central domain variants are predicted to affect assembly of the dimeric unit: E217D, I255T, G271R, M306V, and R317Q. The V303I variant is an exception in that the valine codon begins with a G that is part of the requisite splice site for exon 8. The mutation to Ile changes this nucleotide to an A and eliminates the 3′ splice site, so an intron is left in the ‘mature’ mRNA. Because of this, there is probably a frameshift that truncates the UGDH protein prematurely. Essentially, V303I is predicted to make no UGDH protein. It is also considered to be null in the R443H heterozygous condition described below for the UDP-sugar binding domain variants.

Relatively few variants exist in the UDP-sugar binding domain of UGDH. This domain binds the UDP-glucose substrate, as well as the UDP-glucuronate product and an endogenous competitive inhibitor, UDP-xylose, which is a critical precursor of proteoglycan production and an important component of normal function by feedback inhibition of the proteoglycan synthesis pathways during development. Two of the variants identified to date, R443H and H449R, are predicted to disrupt proper folding of the domain, which has also been shown to affect contacts between dimeric units and is generally incompatible with UGDH catalytic activity. An additional variant, R442W, is found in a heterozygous patient with the R443H variant. Substitution of Arg442 with Trp is predicted to disrupt both the local folded stability of the UDP-sugar binding domain and the binding of the UDP-sugars, since that Arg is needed to form hydrogen bonds directly to the ribose hydroxyl groups.

## Summary and conclusions

UGDH-related disorders form a specific subtype of congenital disorders of glycosylation that exhibit a spectrum of symptoms and variable response to standard treatments for epileptic encephalopathy and/or cardiac valve defects. It is probable that early molecular detection of UGDH variants can provide better individualized care and limit the severity of developmental impairment in many patients. We have comprehensively reviewed the functional data available in the context of clinical symptoms and found limited correlation of disease severity with specific UGDH variants. However, it is worth emphasizing that the hallmark of UGDH-related disorders is a disruption to cellular motility and differentiation that occurs during embryogenesis and throughout subsequent early tissue morphogenic milestones. The distinction of severity is likely individually affected by environmental exposures as well. A deeper understanding of these mechanisms is needed to define new treatment strategies that may be possible through gene therapy.

**Perspectives**

- UDP-glucose dehydrogenase (UGDH)-related disorders are an understudied subset of congenital disorders of glycosylation that have been shown to lead to heart valve defects, developmental epileptic encephalopathy, and dystroglycanopathy, which are devastating health conditions that often require lifelong care.
- Clinical and molecular research on UGDH-related disorders has revealed a general pattern for inheritance and a spectrum of disease severity that is determined partially by the location and nature of the UGDH variants. Analysis of structural implications for each reported UGDH variant provides insights into the regions of the enzyme most affected by sequence variation.
- The expanding scope of UGDH-related disorders implores greater use of whole-exome sequencing in patient diagnosis and individualized strategies for therapy, which, in the future, will benefit from understanding when and how gene therapy approaches will best benefit the individual disease.

## Abbreviations

CM: congenital microcephaly
CNS: central nervous system
DEE: developmental and epileptic encephalopathy
GAG: glycosaminoglycan
UGDH: UDP-glucose dehydrogenase
WT: wildtype
